# Modified early intensive and treat-and-extend regimen of anti-vascular endothelial growth factor for diabetic macular edema in Taiwan

**DOI:** 10.1038/s41598-023-43931-z

**Published:** 2023-11-07

**Authors:** Jui-Feng Chou, Jian-Sheng Wu, Yi-Ling Chen, San-Ni Chen

**Affiliations:** 1 Department of Ophthalmology, Changhua Christian Hospital, No. 135, Nanxiao Street, Changhua City, Taiwan; 2 Department of Post-Baccalaureate Medicine, College of Medicine, National Chung Hsing University, Taichung, Taiwan; 3 Surgery Clinical Research Center, Changhua Christian Hospital, Changhua, Taiwan; 4 Department of Ophthalmology, Eye Center, China Medical University Hospital, No. 2, Yude Road, Taichung, Taiwan; 5 Department of Ophthalmology, China Medical University, Taichung City, Taiwan

**Keywords:** Diabetes, Diabetes complications, Retinal diseases

## Abstract

Given the rising prevalence of patients with diabetes and increasing treatment burden for patients with vision-threatening diabetic macular edema (DME), we aimed to explore the efficacy of modified early intensive and treat-and-extend regimen of anti-vascular endothelial growth factor (VEGF) therapy under the Taiwan National Insurance Bureau reimbursement policy. We obtained data on 69 eyes treated with initial 4-monthly intravitreal injections of aflibercept or ranibizumab, plus individualized treat-and-extend regimen. At 12 months, the mean (SD) change in LogMAR best corrected visual acuity from baseline was − 0.28 (0.31) in all eyes, while that in the aflibercept and ranibizumab groups were − 0.30 (0.34) and − 0.25 (0.28), respectively. Central retinal thickness decreased by 137.2 (122.4) in all eyes, 138.1 (134.2) in the aflibercept group, and 136.2 (110.9) in the ranibizumab group. Additionally, the aflibercept group had a lower mean number of injections than the ranibizumab group (8.5 vs. 8.7). The last extended dosing interval of > 12 weeks was 31.0% and 16.7% of the eyes in the aflibercept and ranibizumab groups, respectively. The modified anti-VEGF regimens effectively managed DME in terms of functional and anatomical outcomes, and efficiently reduced the healthcare burden by reducing the number of injections and extending treatment intervals within 12 months.

## Introduction

Diabetes mellitus is a worldwide epidemic, with approximately 537 million adults living with diabetes as of 2021. Owing to the continued global increase in diabetes prevalence, the population is predicted to rise to 783 million by 2045^1^. Diabetic retinopathy, one of the systemic microvascular comorbidities, develops over time and gradually leads to visual impairment. Diabetic macular edema (DME), which is a vision-threatening stage of diabetic retinopathy, is the major cause of vision loss among patients with diabetes. By 2045, the number of adults worldwide with clinically-significant DME is projected to increase to 28.61 million^2^.

DME is characterized by the alteration of blood-retina barrier, in which pericyte loss and vascular endothelial cell–cell junction breakdown result in leakage and accumulation of fluid in the retina. The abnormalities of metabolic pathways induced by hyperglycemia lead to increased oxidative stress and inflammation, which in turn contribute to DME development^3,4^. Various cytokines and chemokines are involved in this pathogenesis, and elevated vascular endothelial growth factor (VEGF) is one of the potent contributors to vascular leakage and macula edema^5,6^. The delivery of anti-VEGF agents into the eye is a revolutionary therapy, especially for center-involving DME. Many evidence-based studies have established the effectiveness of anti-VEGF therapy in the treatment of DME; however, the optimal frequency of injection and the duration of the treatment course remain controversial. According to the expert consensus in Taiwan, anti-VEGF therapy should be considered the first-line treatment for patients with center-involving DME. Additionally, early intensive anti-VEGF therapy, comprising at least 3 monthly doses, is strongly recommended. The selection of anti-VEGF agents should involve considering baseline visual acuity (VA) and central retinal thickness (CRT). The primary goal of DME management is to improve functional visual outcomes and reduce anatomical edematous macula while minimizing treatment burden^7^.

With the increasing population of patients with diabetes, the healthcare burden for patients with DME is bound to increase. According to the National Health Insurance (NHI) database from 2004 to 2009, the direct medical cost for DME is three times higher than the average national health expenditure per person in Taiwan^8^. According to Taiwan's NHI policy, only a total of 8 lifetime anti-VEGF injections per eye are reimbursed by healthcare insurance. In clinical practice, ophthalmologists in Taiwan generally start with an early intensive 3 to 5 injections during the initial phase to improve the patient’s VA and promote adherence. Subsequently, an individualized injection in pro re nata (PRN) strategy is employed during the follow-up phase to maintain stable VA and CRT. However, given limited healthcare resources, administering up to 5 intensive anti-VEGF injections during the initial phase may be impractical, as it restricts therapeutic flexibility and planning for ophthalmologists during the follow-up phase.

Considering the rising global prevalence of diabetes and associated visual complications of DME, as well as the limited healthcare resources, it is crucial to ideally adjust the therapeutic strategy of anti-VEGF therapy to optimize clinical care for DME. This study was the first of its kind to investigate the effectiveness of early intensive 4 anti-VEGF injections combined with an individualized treat-and-extend (T&E) strategy for the management of DME.

## Results

Patient demographics and disease characteristics are presented in Table 1. A total of 69 eyes belonging to 54 patients with DME were enrolled in the study. Among them, 36 eyes of 29 patients were treated with aflibercept, while 33 eyes of 25 patients were treated with ranibizumab. Overall, the patients’ mean age was 63.5 years, and 53.7% of the patients were females. Baseline demographics were similar in the two subgroups. The baseline mean (SD) best corrected visual acuity (BCVA) in LogMAR score was 0.66 (0.45) and the baseline mean (SD) CRT was 446.6 (142.2) μm for all eyes. The aflibercept group had poorer baseline vision and higher baseline CRT compared with the ranibizumab group; however, there were no significant differences in baseline visual and anatomical characteristics.

**Table 1:** Baseline demographics and disease characteristics.

	Overall (N = 54)	Aflibercept group (N = 29)	Ranibizumab group (N = 25)	P value*
Mean age, years (SD)	63.5 (10.9)	62.7 (10.6)	64.5 (11.4)	0.455
Female, n (%)	29 (53.7)	14 (48.3)	15 (60.0)	0.389
Laterality, OD:OS	35:34	18:18	17:16	0.900
Phakia:Pseudophakia	45:24	23:33	22:11	0.367
Mean HbA1c, % (SD)	7.5 (1.5)	7.6 (1.8)	7.3 (1.2)	0.561
Mean baseline BCVA, LogMAR (SD)	0.66 (0.45)	0.72 (0.44)	0.59 (0.45)	0.104
Mean baseline CRT, µm (SD)	446.6 (142.2)	476.1 (169.1)	411.3 (91.7)	0.209
Mean duration of treatment, months (SD)	10.5 (2.8)	10.4 (3.0)	10.6 (2.6)	0.983

*BCVA* best-corrected visual acuity, *CRT* central retinal thickness, *HbA1C* hemoglobin A1C, *LogMAR* logarithm of the minimal angle of resolution, *OD* right eye, *OS* left eye. *Statistical comparison between aflibercept group and ranibizumab group.

**Figure 1:**
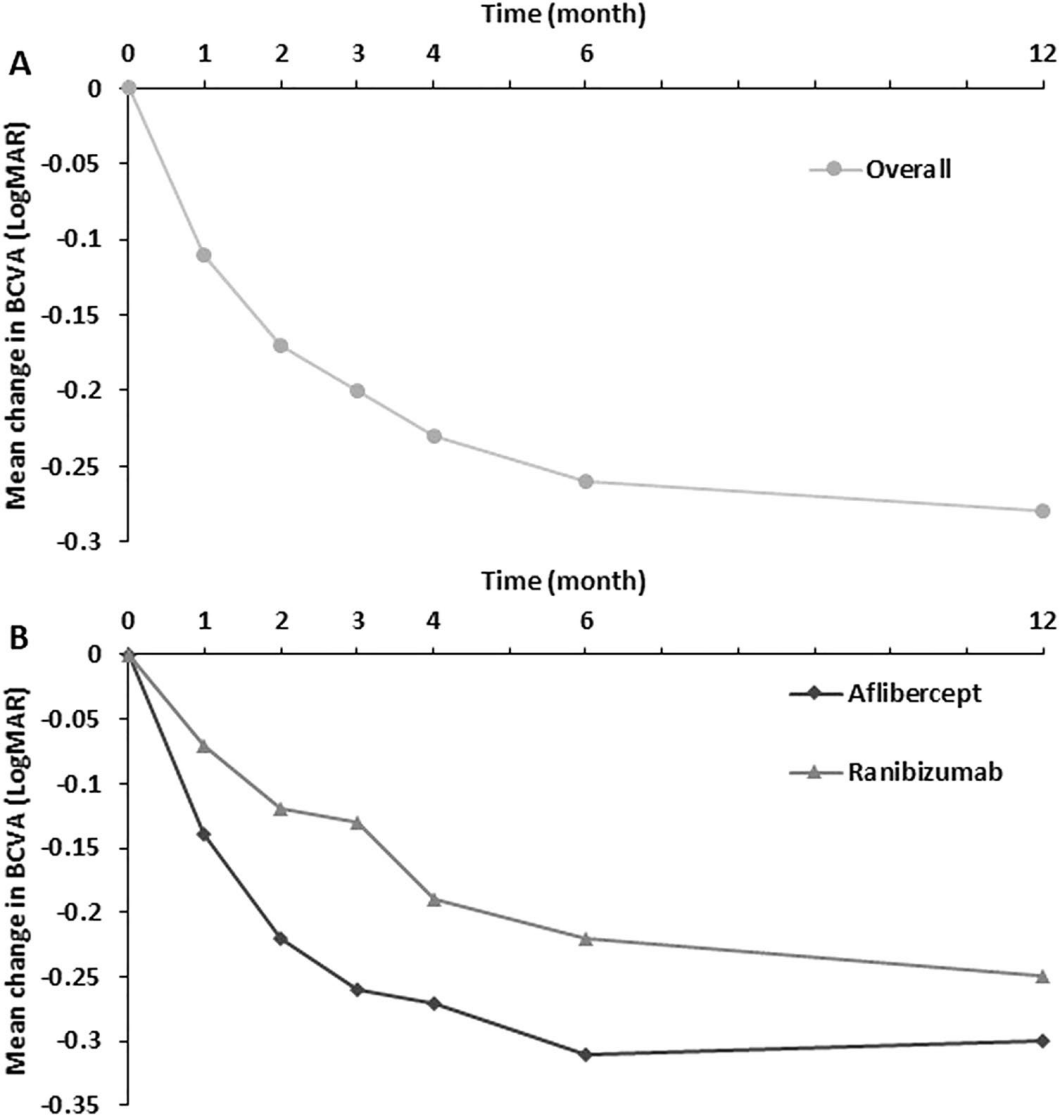
Mean change in BCVA from baseline of study eyes. The BCVA were significantly improving in all eyes (**A**) and within each subgroup (**B**) over the course of 12 months, respectively. However, there was no significant difference between aflibercept group and ranibizumab group at each time point (P > 0.05, dataset shown in Supplementary Table S2).

The mean (SD) change in BCVA from baseline at month 12 was − 0.28 (0.31) in all eyes. In subgroup analysis, it was − 0.30 (0.34) in the aflibercept group and − 0.25 (0.28) in the ranibizumab group. Over the course of 12 months, there was a significant gradual improvement in visual acuity in all eyes and within each subgroup (Fig. 1; Supplementary Table S1). The mean improvement in the visual acuity over time was more apparent in the aflibercept group than in the ranibizumab group, especially within the initial 4 months, although the differences were not statistically significant (P > 0.05 at each time point, Supplementary Table S2).

**Figure 2:**
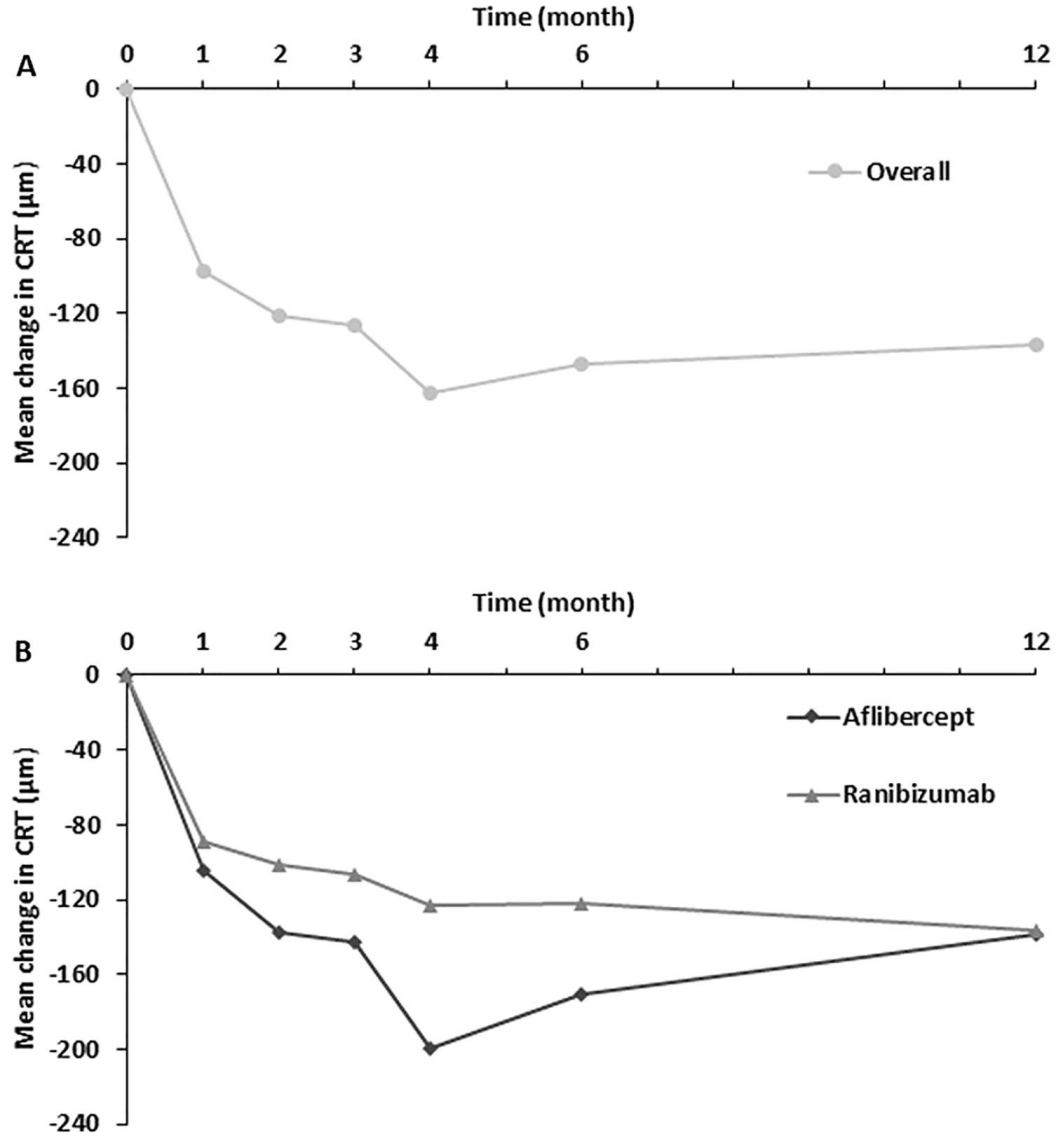
Mean change in CRT from baseline of study eyes. The CRT were significantly decreasing in all eyes (**A**) and within each subgroup (**B**) over the course of 12 months, respectively. However, there was no significant difference between aflibercept group and ranibizumab group at each time point (P > 0.05, dataset shown in Supplementary Table S4).

At the month 12 visit, the mean (SD) of CRT decreased from baseline by 137.2 (122.4) μm in all eyes. Within the subgroup analysis, it decreased by 138.1 (134.2) μm in the aflibercept group and 136.2 (110.9) μm in the ranibizumab group. For the mean change in CRT over the course of 12 months, the CRT significantly decreased gradually in all eyes and within each subgroup (Fig. 2; Supplementary Table S3). Comparatively, the mean reduction in CRT was greater in the aflibercept group than in the ranibizumab group; however, there was no significant difference over 12 months (P > 0.05 at each time point, Supplementary Table S4).

In the subgroup analysis evaluating the efficacy of early intensive therapy followed by a T&E strategy of anti-VEGF injections for managing DME, both the total number of injections within 1 year and the last extended dosing interval are representative indicators. Given the possibility of loss to follow-up of patients due to various reasons in real-world settings, only patients who were completely followed for 1 year were evaluated. In this analysis, the total number of eyes was 29 in the aflibercept group and 24 in the ranibizumab group. The mean (SD) number of injections was 8.5 (1.3) in the aflibercept group and 8.7 (0.9) in the ranibizumab group.

**Figure 3:**
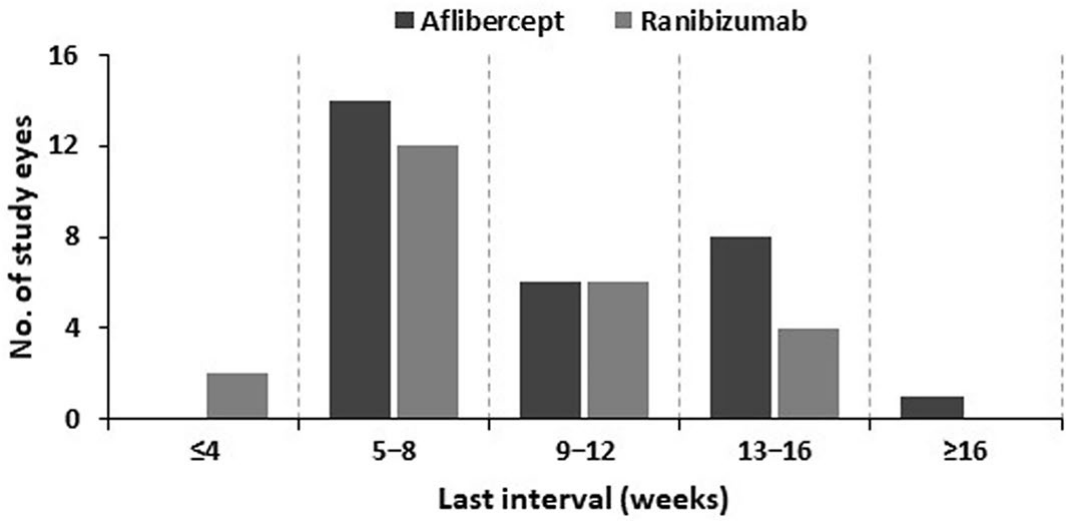
Distribution of last extended dosing intervals in the aflibercept group and ranibizumab group.

The last extended dosing intervals were less than 4 weeks in 0.0% (n = 0), between 5 and 8 weeks in 48.3% (n = 14), between 9 and 12 weeks in 20.7% (n = 6), between 13 and 15 weeks in 27.6% (n = 8), and more than 16 weeks in 3.4% (n = 1) in the aflibercept group. In the ranibizumab group, the last extended dosing intervals were less than 4 weeks in 8.3% (n = 2), between 5 and 8 weeks in 50.0% (n = 12), between 9 and 12 weeks in 25.0% (n = 6), between 13 and 15 weeks in 16.7% (n = 4), and more than 16 weeks in 0.0% (n = 0) (Fig. 3). The distribution of last extended dosing intervals demonstrated that the dosing intervals of patients with DME treated with aflibercept could be extended longer than those treated with ranibizumab.

## Discussion

Anti-VEGF agents have been established as the first-line treatment for DME both in Taiwan and the international community^7,9,10^. Among several commercially available anti-VEGF agents, those currently available in Taiwan include aflibercept (Eylea) and ranibizumab (Lucentis), both of which are eligible for reimbursement, and off-label bevacizumab (Avastin). This real-world study was the first to investigate the efficacy of the modified early intensive anti-VEGF injections combined with T&E strategy in managing DME. Both aflibercept and ranibizumab demonstrated effectiveness in significantly improving functional and anatomical outcomes. Furthermore, the combined T&E dosing regimens proved to be efficient in addressing the treatment burden.

Most randomized clinical trials investigating DME treatment using anti-VEGF administered at least 3 monthly doses during the initial phase^11–15^. The 1-year outcomes of the phase 2 DA VINCI study showed that a more intensified anti-VEGF regimen (i.e., 2 mg every 4 weeks vs. 2 mg every 8 weeks) after the 3 monthly loading injections was associated with greater improvements in BCVA and CRT^16^. The subsequent VIVID/VISTA study, which employed 5 monthly loading injections, demonstrated continual functional and anatomical improvement following the 4th and 5th loading injections^14,17^. Consequently, early intensive anti-VEGF therapy has been recommended to significantly improve vision, providing an advantage in promoting patient adherence in a clinical setting. However, a total of 8 anti-VEGF agents per eye lifetime are eligible for reimbursement based on the NHI policy in Taiwan. Under limited healthcare resources, administering up to 5 monthly loading injections during the initial phase is infeasible because it restricts treatment flexibility during the follow-up phase. Therefore, an anti-VEGF regimen with 4 monthly loading injections during the initial phase is a reasonable adjustment, considering both the effective treatment and limited healthcare resources. In our study, both the overall and each subgroup analysis demonstrated continual functional and anatomical improvement following the 4th loading injection.

The T&E strategy is a cost-effective approach intended to reduce treatment burden by minimizing the number of hospital visits and injections while achieving stable disease activity. It is an individualized treatment algorithm for the management of retinal diseases with anti-VEGF agents, in which the interval between treatments is promptly adjusted based on disease activity at each visit^18^. The T&E regimen with anti-VEGF agents, mainly applied to treat neovascular age-related macular degeneration, is gaining international popularity among retina physicians^19^. Regarding the treatment of DME, several randomized controlled trials have demonstrated the efficacy of the T&E strategy^20–22^. A systematic review further confirmed that, compared with fixed or PRN regimens, the T&E regimen provided noninferior functional and anatomical outcomes at 12 and 24 months, despite a higher number of injections than the PRN regimen at 12 months^23^. While the T&E regimen may not reduce the number of injections compared to PRN regimen, it holds the potential to reduce the frequency of patient visits, thereby alleviating treatment burden^24^. In our analysis of patients with DME, the modified early intensive 4 loading injections and T&E regimen of anti-VEGF agents significantly improved both functional and anatomical outcomes within 12 months. The visual benefits of such efficient anti-VEGF therapy for DME in real-world setting were comparable to those observed in prospective controlled studies^20–22^. Additionally, both the total number of injections and the last extended dosing interval indicate the efficacy of anti-VEGF therapy. In our study, the results among each subgroup demonstrated a considerable efficiency of anti-VEGF agents in the treatment of DME.

As aflibercept has a greater blocking potency and stronger affinity for VEGF than ranibizumab^25,26^, it is expected to have a superior effect, require fewer injections and allow for more extended dosing interval in the treatment of DME. The head-to-head Protocol T study demonstrated that aflibercept was associated with greater vision improvement at 1 year among eyes with baseline VA less than 20/50, compared with ranibizumab or bevacizumab^15^. However, the improvement at 2 years was comparable between aflibercept and ranibizumab^27^. Although there was no statistical significance, the comparative analysis of the subgroups in this study revealed that aflibercept exhibited a trend towards better functional and anatomical improvements. Aflibercept also required fewer injections, and had relatively longer extended treatment interval compared to ranibizumab within 12 months. To some extent, the superior efficacy of aflibercept in this study was comparable to that observed in the Protocol T study^15^. Furthermore, it was also reported that switching to aflibercept for persistent DME with suboptimal response to ranibizumab provided significant anatomic improvements^28^ and significant improvements in BCVA^29^.

This study had certain limitations. First, it was a retrospective and interventional study without a control group, such as an initial 3 or 5 monthly injection combined with either fixed or PRN anti-VEGF regimens. Secondly, the sample size was small and some proportion of eyes were lost to follow-up (7/36 in the aflibercept group; 9/33 in the ranibizumab group) within the 12-month period. This resulted in insufficient power, which limited our ability to perform subgroup analysis based on baseline BCVA and CRT and may have made it difficult to detect significant differences between subgroups, as demonstrated in the Protocol T study^15^. Additionally, the relatively short 12-month follow-up period may have limited the T&E regimen’s ability to demonstrate its full effect in reducing the injection burden. The average number of injections per year would be expected to decrease as the follow-up period is lengthened. Previous studies mostly reported the clinical practice of anti-VEGF therapy in the treatment of DME in Taiwan. The strength of this study lies in the fact that it is the first study to explore the efficacy of early intensive 4 monthly injections combined with a T&E anti-VEGF regimen in a real-world setting, aiming to effectively stabilize disease activity and efficiently reduce the healthcare burden in the treatment of DME.

In conclusion, both aflibercept and ranibizumab were effective in improving functional and anatomical outcomes in eyes with DME under the reimbursement policy of NHI in Taiwan. Considering effective treatment and limited healthcare resources, the modified early intensive and T&E anti-VEGF regimen is practically applicable in the clinical care of patients with DME. However, there is a need for further prospective studies to compare the different dosing regimens to maximize the efficacy in the management of DME.

## Methods

### Patient selection

This retrospective, interventional cohort study utilized longitudinal data from medical records of patients with DME who were treated with intravitreal anti-VEGF therapy at the ophthalmology department of Changhua Christian Hospital between January 2017 and December 2018. The study protocol was approved by the institutional review boards of Changhua Christian Hospital (approval number: 230120), and the study was conducted in accordance with the tenets of the Declaration of Helsinki. Informed consent was obtained from all participants. Medical records were reviewed to identify patients who met the following criteria: (1) age > 18 years; (2) diagnosis of center-involving DME (CRT ≥ 300 μm on optical coherence tomography); and (3) decimal BCVA between 0.5 and 0.05 (Snellen visual acuity); and (4) HbA1c < 10%, all of which corresponded to NHI eligibility criteria for the reimbursement of anti-VEGF therapy of aflibercept or ranibizumab. The exclusion criteria were: (1) history of vitreoretinal surgery in the eye regardless of time; (2) intraocular surgery, focal/grid laser photocoagulation, or anti-VEGF treatment in the eye within 90 days prior to enrollment; (3) ocular disorders that may contribute to macular edema or affect VA. Patients with DME who were treated with combined early intensive 4 monthly doses and T&E of anti-VEGF regimen were enrolled in this study.

### Anti-VEGF regimen

The eye under study received initial 4 monthly intravitreal injections of 2 mg aflibercept or 0.5 mg ranibizumab. Subsequently, the anti-VEGF injection intervals were then adjusted based on the T&E strategy. In cases where ocular conditions were stable, including BCVA variability less than one line of Snellen's chart, CRT variability less than 10%, or a CRT less than 320 μm, the injection interval was extended 2 to 4 weeks. However, if the ocular condition was deemed unstable, the interval was shortened (a minimum of 4 weeks).

### Assessment

Medical records, including BCVA, CRT, extended dosing interval and the total number of injections within 1 year, were extracted from our institutional database. The BCVA measured using Snellen's chart was converted to the logarithm of the minimum angle of resolution (LogMAR) for analysis. The CRT was obtained using spectral domain optical tomography (SD-OCT) (Spectralis, Heidelberg Engineering, Heidelberg, Germany) images at each visit. The measurements were performed on the 1-mm Early Treatment Diabetic Retinopathy Study (ETDRS) circle centered on the fovea using the mapping protocol of the SD-OCT software.

### Statistical analysis

All statistical analyses were performed using SPSS version 26 software (SPSS Inc., Chicago, Illinois, USA). Descriptive statistics were used to summarize patient demographic and disease characteristics. Chi-square test was conducted to analyze categorical variables. We performed Shapiro–Wilk test for normality. For non-normally distributed data, we performed a Wilcoxon signed-rank test to compare the changes in BCVA and CRT with baseline of each eye, and a Mann–Whitney U test to compare differences in the baseline and mean changes of BCVA and CRT in the subgroups. A P value < 0.05 was considered statistically significant.

### Supplementary Information

Supplementary Tables.

## Data Availability

The datasets used and/or analyzed during the current study are available from the corresponding author on reasonable request.
