# Supplementary material for: Modified early intensive and treat-and-extend regimen of anti-vascular endothelial growth factor for diabetic macular edema in Taiwan
Source: Sci Rep. 2023 Nov 7;13:19349. doi: 10.1038/s41598-023-43931-z (PMC10630418; doi:10.1038/s41598-023-43931-z)
Supplement: Supplementary file 1 — Supplementary Tables. [file 41598_2023_43931_MOESM1_ESM.docx]

| BCVA, LogMAR (SD) | Overall | p value | Aflibercept group | p value | Ranibizumab group | p value |
| --- | --- | --- | --- | --- | --- | --- |
| Baseline | 0.66 (0.45) |  | 0.72 (0.44) |  | 0.59 (0.45) |  |
| 1M | 0.54 (0.41) | 0.002 | 0.58 (0.41) | 0.028 | 0.52 (0.40) | 0.019 |
| 2M | 0.49 (0.30) | <0.001 | 0.50 (0.35) | <0.001 | 0.47 (0.43) | 0.055 |
| 3M | 0.46 (0.41) | <0.001 | 0.46 (0.32) | <0.001 | 0.46 (0.49) | 0.018 |
| 4M | 0.47 (0.33) | <0.001 | 0.49 (0.30) | 0.001 | 0.43 (0.36) | 0.003 |
| 6M | 0.38 (0.30) | <0.001 | 0.39 (0.28) | <0.001 | 0.35 (0.32) | <0.001 |
| 12M | 0.34 (0.28) | <0.001 | 0.39 (0.26) | <0.001 | 0.29 (0.29) | <0.001 |

**Table S1.** The mean BCVA at baseline and each time point. Statistical comparison between each time point and baseline within overall patients or each subgroup, respectively.

| Mean change in BCVA, LogMAR (SD) | Overall | Aflibercept group | Ranibizumab group | p value |
| --- | --- | --- | --- | --- |
| 1M | -0.11 (0.30) | -0.14 (0.36) | -0.07 (0.21) | 0.747 |
| 2M | -0.17 (0.34) | -0.22 (0.32) | -0.12 (0.40) | 0.150 |
| 3M | -0.20 (0.35) | -0.26 (0.30) | -0.13 (0.40) | 0.212 |
| 4M | -0.23 (0.34) | -0.27 (0.36) | -0.19 (0.32) | 0.520 |
| 6M | -0.26 (0.32) | -0.31 (0.38) | -0.22 (0.26) | 0.450 |
| 12M | -0.28 (0.31) | -0.30 (0.34) | -0.25 (0.28) | 0.504 |

**Table S2.** Mean change in BCVA from baseline. Statistical comparison between Aflibercept and Ranibizumab group at each time point.

| CRT, µm | Overall | p value | Aflibercept group | p value | Ranibizumab group | p value |
| --- | --- | --- | --- | --- | --- | --- |
| Baseline | 446.6 (142.2) |  | 476.1 (169.1) |  | 411.3 (91.7) |  |
| 1M | 352.2 (80.8) | <0.001 | 371.4 (92.9) | <0.001 | 328.3 (55.6) | <0.001 |
| 2M | 325.7 (68.9) | <0.001 | 338.9 (67.8) | <0.001 | 309.9 (68.0) | <0.001 |
| 3M | 320.0 (66.9) | <0.001 | 332.9 (67.5) | <0.001 | 304.5 (63.8) | <0.001 |
| 4M | 305.4 (80.7) | <0.001 | 310.7 (63.4) | <0.001 | 299.5 (98.1) | <0.001 |
| 6M | 296.7 (62.7) | <0.001 | 297.3 (60.7) | <0.001 | 296.0 (66.1) | <0.001 |
| 12M | 280.7 (44.1) | <0.001 | 294.9 (45.8) | <0.001 | 263.9 (36.5) | <0.001 |
| Final | 285.9 (54.3) | <0.001 | 290.3 (53.1) | <0.001 | 280.6 (56.1) | <0.001 |

**Table S3.** The mean CRT at baseline and each time point. Statistical comparison between each time point and baseline within overall patients or each subgroup, respectively.

| Mean change in CRT, µm (SD) | Overall | Aflibercept group | Ranibizumab group | p value |
| --- | --- | --- | --- | --- |
| 1M | -97.6 (102.9) | -104.7 (117.3) | -88.7 (82.9) | 0.916 |
| 2M | -120.9 (133.0) | -137.2 (155.6) | -101.4 (98.5) | 0.648 |
| 3M | -126.6 (146.3) | -143.1 (177.7) | -106.8 (95.7) | 0.969 |
| 4M | -163.0 (175.6) | -199.9 (198.6) | -122.8 (140.0) | 0.219 |
| 6M | -147.6 (154.7) | -170.2 (188.4) | -122.4 (103.3) | 0.848 |
| 12M | -137.2 (122.4) | -138.1 (134.2) | -136.2 (110.9) | 0.915 |

**Table S4.** Mean change in CRT from baseline. Statistical comparison between Aflibercept and Ranibizumab group at each time point.
